# The origins of music in auditory scene analysis and the roles of evolution and culture in musical creation

**DOI:** 10.1098/rstb.2014.0089

**Published:** 2015-03-19

**Authors:** Laurel J. Trainor

**Affiliations:** 1 Department of Psychology, Neuroscience and Behaviour, McMaster University, Hamilton, Ontario, Canada; 2 McMaster Institute for Music and the Mind, McMaster University, Hamilton, Ontario, Canada; 3 Rotman Research Institute, Baycrest Hospital, Toronto, Ontario, Canada

**Keywords:** music, evolution, auditory scene analysis, entrainment, pitch, metre

## Abstract

Whether music was an evolutionary adaptation that conferred survival advantages or a cultural creation has generated much debate. Consistent with an evolutionary hypothesis, music is unique to humans, emerges early in development and is universal across societies. However, the adaptive benefit of music is far from obvious. Music is highly flexible, generative and changes rapidly over time, consistent with a cultural creation hypothesis. In this paper, it is proposed that much of musical pitch and timing structure adapted to preexisting features of auditory processing that evolved for auditory scene analysis (ASA). Thus, music may have emerged initially as a cultural creation made possible by preexisting adaptations for ASA. However, some aspects of music, such as its emotional and social power, may have subsequently proved beneficial for survival and led to adaptations that enhanced musical behaviour. Ontogenetic and phylogenetic evidence is considered in this regard. In particular, enhanced auditory–motor pathways in humans that enable movement entrainment to music and consequent increases in social cohesion, and pathways enabling music to affect reward centres in the brain should be investigated as possible musical adaptations. It is concluded that the origins of music are complex and probably involved exaptation, cultural creation and evolutionary adaptation.

## 1. Introduction

The origins of complex behaviours and cognitive abilities are of great interest in the field of evolutionary psychology [1–3]. The origin of musical behaviour is a particularly interesting example because there is currently no agreement as to whether music was an evolutionary adaptation or a cultural creation. Although the universality and early developmental emergence of musical behaviour are consistent with it being an evolutionary adaptation, its adaptive value is not agreed upon or, indeed, obvious [4–6]. A number of potential evolutionary pressures for music have been proposed, and evidence for them discussed (reviewed [4–8]), such as sexual selection [9,10], social bonding and group cohesion [11–13], regulating infant arousal and behaviour [14–17], aiding cooperative labour through rhythmic coordination, perceptual and motor practice or skill development [18], conflict resolution, safe time passing, and as a memory aid for preserving important cultural information across generations [7]. On the other hand, it has also been proposed that music is not an evolutionary adaptation, but rather a cultural creation that can stimulate pleasure centres in the brain (e.g. ‘auditory cheesecake’ hypothesis [19]), a by-product of the evolution of language (e.g. [19,20]) or a culturally created ‘transformative technology’ that affects our experience of the world [21].

In this paper, it is argued that these seemingly opposing views of musical origins—evolutionary adaptation versus a cultural creation—can be reconciled by going beyond simple notions of adaptive processes. Specifically, musical behaviour rests on the interaction of adaptations shaped by natural selection and social–cultural forces. A major question is whether adaptations were selected to enhance music specifically, or whether the evolutionary pressures were for other traits or capacities related to auditory perception, cognition and motor skills which, once in place, made music possible. According to the former view, the benefits of musical behaviour drove the evolutionary adaptations; according to the latter view, music is a cultural creation that was moulded to existing brain structures and capacities that evolved under other pressures.

Evolutionary biologists describe an adaptation as a trait that has been shaped or modified by natural or sexual selection through particular gene-promoting effects [22]. An adaptationist hypothesis is therefore a claim about the effects that, in the ancestral past, were favoured by natural or sexual selection and contributed to shape current structure or operation. It is not a claim about current selection pressures that may or may not be maintaining it in populations. Consequently, the study of adaptation is largely an historical science [23]. In evolutionary biology, the term *function* is reserved for an effect that contributed to the shaping or modification of an adaptation by natural selection.

It is possible for some traits to take on new beneficial effects, without being modified by selection for those effects. Such traits are called exaptations for these effects [24]. The distinction between an exaptation and an adaptation rests on whether or not the trait has been modified or shaped by selection specifically to facilitate a beneficial effect. For instance, the contour feathers of birds probably evolved first in small dinosaurs for a thermoregulatory function by providing a flat surface over which wind could pass without disturbing the warm air trapped close to the body [1]. But the structural organization of contour feathers also proved useful for facilitating flight. However, natural selection subsequently lengthened and stiffened the contour feathers located on the forelimbs and tails specifically because of the flight facilitating effect. Thus, contour feathers were first adapted to thermoregulation, then exapted to flight, and some contour feathers underwent secondary adaptation for flight. Note that when a trait does not exhibit any specific modification for a beneficial effect, that effect cannot be said to be a function of the trait. Only adaptations have functions. It would be appropriate to say that facilitating flight is the function of the lengthened and strengthened feathers on the wings and tails of birds. However, it would not be appropriate to say that flight is the function of contour feathers on the abdomen, unless specific modification for promoting flight could be demonstrated.

Finally, some traits may not be directly favoured by natural selection, but are inextricably tied (by genetic or developmental constraints) to traits that were the outcome of selection. Such traits are termed *by-products* or *spandrels* [25], after the triangular-shaped spaces between architectural arches. It is impossible to build a row of arches without producing these spaces, although there was no intent to do so. Spandrels can have neutral, beneficial or even harmful effects. If a spandrel has a beneficial effect, then it may also qualify as an exaptation for that effect, provided it has not been modified by selection to promote that effect.

The evaluation of evolutionary hypotheses is difficult, as has been reviewed by others [1,22,26]. Musical behaviour does have a number of features consistent with the idea that it was in part an evolutionary adaptation, such as an ancient origin (bone flutes date to at least 36 000 years ago and vocal music probably much earlier [27,28]), universality across human cultures, early ontogenetic emergence without formal instruction, similarities (as well as variations of course) in pitch and rhythmic structures across musical systems, connections between auditory rhythms and entrained movement across cultures, the universal proclivity to respond emotionally to music, and use in ritual and social engagement across societies (e.g. [4,5,7,11]).

On the other hand, the origins of complex cognitive abilities, such as music and language, that are highly flexible, generative and whose manifestations change rapidly over time pose particular challenges for evolutionary theories (see [29] for a discussion of this question with respect to language). Just as there are many languages, there are many musical systems. Because they carry less conventional sound–meaning mappings, musical systems may change even more rapidly than languages. When different musical systems come into contact, new musical styles can readily emerge. For example, regional folk songs and jazz have influenced classical music, and new styles have emerged from fusions between jazz and rock music. Given that an exclusively evolutionary explanation for the origins of music would have difficulty explaining the variety of musical styles and the rapidity of musical change, there would appear to be a strong cultural component to musical origins.

In the case of music, the evolutionary question has typically been posed as whether musical behaviour fits into one of three evolutionary processes: (i) *adaptation*. There were selection pressures on the nervous system specifically for musical behaviour, such as increased group social cohesion, which led to increased survival, or signalled increased fitness in mate selection. (ii) *Exaptation*. For example, the evolution of language might be an adaptation, leading to survival benefit for individuals in groups that could use language to communicate specific information; the auditory, memory and cognitive adaptations needed for language also enabled music, which has survived over the long term because it enriches us culturally, even though music was not directly selected for. (iii) *Spandrel*. For example, the auditory system evolved under pressure to better sense danger in the environment, and pleasure centres in the brain evolved in order to motivate behaviours needed for survival and procreation; music just happens to use the auditory system in ways that activate pleasure centres, but the auditory system has not been modified by selection to do so.

This paper takes a somewhat different approach. Rather than starting with the question of what functions music has or had in the past, and therefore what adaptive pressures might have been involved in the emergence of music, this paper begins by examining the structure of music itself and determining what capabilities are needed for the perception and processing of music. The origins of these capabilities are then examined in light of developmental and cross-species comparisons to determine whether the capabilities in question evolved for functions other than music. Only capabilities necessary for music that did not obviously evolve for any other function are considered as candidates for music-specific adaptations. The three processes of adaptation, exaptation and spandrels are often intertwined, particularly for the emergence of complex traits and complex cognitive abilities (see [1] for a detailed and insightful discussion). In this paper, it is argued that all three processes were probably involved in the emergence of critical structures necessary for music, but that for the most part this occurred through selection pressures for non-musical functions. Those traits, or inextricably linked traits, may have then enabled musical or protomusical behaviours as cultural creations. However, even if music was largely a cultural creation, it is also possible that to the extent that music itself was beneficial, further music-specific adaptation occurred subsequently. Indeed, for the emergence of something as complex as music, there may have been a number of iterations of adaptive, exaptive and cultural processes.

Music involves many aspects, such as pitch perception, time perception, pattern perception, rhythm or metrical perception, emotional responses, memory, sound production and social consequences. It is possible, indeed likely, that different adaptive pressures and histories of adaptive and exaptive processes applied to these different aspects, and that in many cases the adaptive pressures were not for music. In the following sections, I will consider pitch-based aspects of music, time- and rhythm-based aspects, and social–emotional aspects. For each, I will consider possible evolutionary origins of particular traits or behaviours necessary for music, and whether there is evidence for music-specific adaptations.

Where available, I examine evidence from ontogenetic development. Ontogenesis is informative, as the early emergence of a trait or ability in development suggests that cultural origins are less likely, or at least that the organism is prepared to learn quickly in that domain. As for cross-species comparisons, in the case of music, it is generally agreed that humans are the only species to produce music [30]. A few other species do engage in music-like behaviours (e.g. some vocal learning birds produce generative vocalizations and some will entrain to musical rhythms [31,32]), but it is particularly revealing that our genetically closest relatives do not engage in musical activity, nor do musical stimuli appear to interest or engage them ([33,34], but see [35]). In any event, neurological structures or processes that play a role in the musical behaviour of humans but are widely conserved across species are likely to originate from adaptive pressure unrelated to music, and to therefore be exaptations or spandrels with respect to music. Conversely, neurological structures or processes unique to humans represent phenotypic modifications that may have arisen by natural selection for behaviours specific to humans, including musical behaviour (i.e. they represent candidate adaptations that should be rigorously scrutinized).

This paper is not intended to provide an exhaustive consideration of the evolutionary and cultural origins of music, but rather presents hypotheses about how adaptive, exaptive and cultural processes may have been involved in some aspects of musical emergence, in the context of a discussion of how to evaluate hypotheses in this domain. The first sections focus on perceptual prerequisites for musical behaviour. In particular, I will argue that much of musical spectral (pitch) and temporal (rhythm and metre) structure rests on adaptations of the auditory system for gathering information about what sounding objects are present in the environment and where they are located, a process termed auditory scene analysis (ASA) [19,36]. Specifically, in §2, I present a brief overview of ASA and discuss the fact that it is phylogenetically old and emerges early in development. In §3, I consider what aspects of musical pitch structure can and cannot be explained by ASA, and in §4, what aspects of musical temporal structure can and cannot be explained by ASA. I argue that, rather than music exhibiting adaptive pressure on the auditory system, it is largely the other way around: pitch and rhythmic structure in music has adapted or conformed to preexisting features of the auditory system. However, there may be some features of music that were evolutionary adaptations, and evidence for these will be considered. In §5, I examine possible adaptive social and emotional aspects of music and consider whether they might have exerted adaptive pressure for enhanced musical perception and production.

## 2. Auditory scene analysis

The most basic functions of perception include determining *what* objects are present in the environment and *where* they are located [37], information that is useful for a wide variety of species. Unlike the visual system, where the relative location of objects in space is related to the spatial pattern of activity on the retina and topographic maps in visual pathways, in the auditory system, sound vibration frequency is encoded along the basilar membrane in the inner ear, and this organization is maintained in tonotopic maps throughout subcortical pathways and into primary auditory cortex. Thus, location must be calculated on the basis of complex cues such as interaural time and intensity differences, and sound filtering properties of the pinna [38]. In the visual system, one object may occlude another object, but the corresponding problem in the auditory system is more complex in that (i) most sounds emitted by objects in the environment contain energy across a wide range of frequencies, so different sounds overlap in frequency content, and (ii) an auditory environment typically contains many simultaneously sounding objects and the sound waves emitted by these objects (and their echoes) are combined in the air and reach the ear as one complex wave. Thus, ASA involves decomposing the sound input into spectrotemporal components (i.e. the frequency content and how it changes over time) and figuring out how many sound sources there are and which components come from which sound sources. This requires segregation of some components as originating from different sources as well as the integration of other components as coming from the same sound source. This determination is not an easy problem to solve, and the auditory system relies on a number of cues [36].

As outlined by Bregman [36], ASA in humans has two aspects, bottom-up automatic parsing of the input, as well as top-down controlled processes, which deploy attention and knowledge of familiar sounds. The cues used by the auditory system in automatic ASA have been studied extensively. They can be grouped into two categories, those related to separating *simultaneous* sound sources (e.g. one person's voice from other voices at a cocktail party, see [39] for a review) and those related to integrating *successive* sounds emitted over time from one object (e.g. integrating successive speech sounds emitted by one talker, or successive notes played by one musical instrument, into a single stream of sound, e.g. [40,41]). Of course, simultaneous and successive processes occur at the same time. For example, in music written for two voices, at any moment in time, the auditory system must determine that there are two voices present, which frequency components (harmonics) belong to each voice, while at the same time following the successive frequency changes within each voice and integrating them into melodic percepts [42].

Bottom-up processes in ASA are sometimes surprisingly opaque to top-down influence [36], suggesting an evolutionarily ancient origin. Indeed, ASA has been identified across many species (see [43] for a review). ASA also emerges early in human development [44–49]. The cues used to accomplish ASA are complex, but a number have been identified and, in some cases, how they interact when in conflict to produce stable percepts has been observed (see [36,50] for reviews). For both simultaneous and successive aspects of ASA, both spectral (frequency) based and temporal (timing) based cues are used. These are discussed in the next sections.

## 3. Spectral analysis and the origins of musical pitch structure

Pitch perception is fundamental to music, raising the possibility that it might have evolved for musical behaviour. However, I will show here that (i) pitch is not given in the stimulus, but derived by the brain and (ii) the perception of pitch is a direct consequence of ASA. Vowel-like vocalizations and musical instrument sounds that are perceived to have a pitch typically have energy at a fundamental frequency, *f*_0_, and at harmonics whose frequencies are at integer multiples of *f*_0_. For example, if *f*_0_ = 100 Hz, the harmonic frequencies will be 200, 300, 400, 500, … Hz. The cochlea in the inner ear is stiffer and wider at one end than the other, causing it to vibrate maximally at different points along its length according to the frequency input in a systematic manner. The vibration of the basilar membrane is transduced into electrical signals in the auditory nerve via the inner hair cells along its length, creating a tonotopic representation that is maintained through subcortical nuclei and into primary auditory cortex. Thus, when a complex sound (i.e. one with several frequency components or harmonics) is presented, the basilar membrane performs a sort of Fourier analysis, decomposing it into its frequency components, which are maintained in separate channels. Additionally, there is a temporal aspect of frequency coding (e.g. [51–53]). Inner hair cells fire at the point of maximal displacement of the basilar membrane, so the timing of populations of neurons also encodes frequency content, and current models of pitch perception combine spectral and temporal cues [54–56]. Accumulating evidence suggests that it is not until information reaches an area just beyond primary auditory cortex on the lateral side of Heschl's gyrus that the spatial frequency and temporal frequency representations are combined and that the frequency content is integrated into a percept of a single sound (auditory object) with a particular pitch and timbre [57–62].

**Figure 1.**
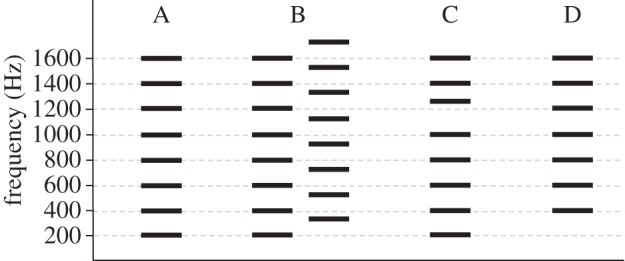
Harmonic structure and determining the number of auditory objects with simultaneous sound inputs. (A) A complex tone with fundamental frequency (*f*_0_) at 200 Hz and harmonics at integer multiples of *f*_0_, which is perceived as a single tone (auditory object) with a pitch of 200 Hz. (B) Two complex tones (sound sources) with *f*_0_s at 200 and 260 Hz and their harmonics. It can be seen that their harmonics overlap in frequency range, so when they simultaneously impinge on the ear, the auditory system must decompose the incoming sound into its frequency components and use its knowledge of harmonic structure to recombine them into representations of the original sound sources. (C) That the brain uses harmonicity to determine the number of auditory objects can be seen by mistuning one harmonic of the 200 Hz complex tone shown in (A). In this case, two tones are heard. The mistuned harmonic is heard as one auditory object and the remaining components, which are all integer multiples of *f*_0_, fuse into a second auditory object. (D) Pitch of the missing fundamental: the brain creates the sensation of pitch as can be seen in that when *f*_0_ is removed from a complex tone stimulus, the perceived pitch remains at *f*_0_.

One might ask why the auditory system decomposes an incoming sound into its frequency components only to reintegrate them once again in cortex. The answer is that the process is necessary for ASA. When two or more sound sources are present in the environment at the same time, and their frequency ranges overlap, the only way to determine which frequency components belong to which sound (or indeed, how many sounds are present) is to decompose the incoming sound wave by frequency and recombine the components according to probable sound sources (figure 1, A and B).

One important cue for determining whether a set of simultaneous frequencies should be integrated into a single percept is whether or not the frequencies are integer multiples of a common fundamental frequency, as this is a common sound structure in human and non-human vocalizations. The perception of pitch is one consequence of this process. That pitch is derived in the brain and not given in the sound input is clearly demonstrated by the phenomenon known as perception of the pitch of the missing fundamental (figure 1, D). Specifically, if the energy at *f*_0_ is removed (and masking noise covers any difference tones created by nonlinearities in the ear), the structure of the harmonics leads to perception of a sound with pitch at *f*_0_, even though there is no energy at that frequency (although timbre will change, of course) [63]. Thus, pitch perception appears to have evolved as a consequence of ASA and not specifically for music. Consistent with this idea, many species perceive the pitch of the missing fundamental (e.g. [64]). In human infants, perception of the pitch of the missing fundamental emerges at around three months of age as auditory cortex matures and supports information processing [65]. Thus, the evidence strongly indicates that pitch perception did not evolve for music but rather was exapted for music. Indeed, it could be considered that, in this case, music conformed to the human auditory system, rather than the other way around, as has been suggested for language [29,66].

Harmonic relations, or their absence, are also used in ASA to separate frequency components into different auditory objects (e.g. [36,67]). For example, if one harmonic of a complex tone is mistuned, it is no longer integrated with the other frequency components and is perceived as a separate auditory object [68] (figure 1, C). The ability to hear two objects when a harmonic is mistuned appears to emerge in human infancy at around the same age as the ability to derive pitch from sounds with missing fundamentals [46,69], consistent with the idea that both are part of the same process of ASA. Music often consists of more than one sound at a time. As with the perception of pitch itself, the ability to perceive multiple simultaneous musical lines appears to be based on the evolution of ASA, again consistent with musical structure being a consequence of the human auditory system rather than music driving the evolution of the auditory system.

Other aspects of musical pitch structure also appear to be a consequence of the structure of the inner ear. For example, the physical properties of the basilar membrane are such that its frequency tuning increases with increasing frequency [70]. Specifically, when two frequencies that differ by less than a *critical band* are presented simultaneously, their vibration patterns interact on the basilar membrane so that they are not cleanly encoded in different tonotopic channels, and it is more difficult to determine which frequencies are present. The size of the critical band increases with increasing frequency up to at least 1000 Hz [71] and probably well beyond [72,73], which means that for lower tones, greater frequency separation is needed in order to clearly perceive the pitches of the tones [74–76]. As discussed above, frequency coding on the basilar membrane, in the form of a tonotopic map, is the first step in ASA because only by separating the frequency components in a sound wave can it be determined which components belong to which auditory objects. Critical bands are a direct result of the nature of physical vibrations on the basilar membrane, so they can be considered a by-product of adaptations for ASA. As Huron [42] points out, music is written with larger pitch differences between, for example, bass and tenor parts than between soprano and alto parts, in a manner that parallels the size of the critical band. It is highly unlikely that music exerted an influence on the evolution of critical band size. Instead, for the pitch content of music to be clear, it must conform to basic constraints of the auditory system that evolved for other functions.

Similarly, when two musical tones are played simultaneously, musicians and non-musicians and even infants encode the pitch of the higher tone better than that of the lower tone [77–79]. Interestingly, this effect also originates in interactions between harmonics during frequency coding on the basilar membrane in the cochlea ([80]; see box 1). Although there are no animal studies on this effect, its peripheral origin suggests that it will probably also be found in other mammals. Musical composition is consistent with this property of sound encoding as seen in the widespread placement of the main melody in the highest pitched voice in polyphonic music. It is highly unlikely that the critical band structure in the inner ear was specifically selected for music. Indeed, the effects of critical band structure on frequency encoding and the high-voice superiority effect are probably spandrels (i.e. non-adaptive consequences) of ASA that in turn affect how music is composed and experienced. That said, it is possible that once critical band structure had evolved, music and/or language exerted additional pressures to sharpen cochlear tuning; consistent with this possibility, it has been estimated that human cochlear tuning is better than that of most other mammals by a factor of two to three [71,73].

Box 1. The high-voice superiority effect for pitch and the low-voice superiority effect for timing of simultaneous tones originate in the cochlea of the inner ear. When two simultaneous tones are presented, as in panel 1*a*, from Marie & Trainor [79], the brain responds more strongly to occasional pitch changes of a semitone (1/12 octave) in the higher than the lower tone as measured by the mismatch negativity (MMN) response of the event-related potential in electroencephalographic (EEG) recordings, but not when each tone is presented separately. When the high tone or the low tone is passed through a computer model of the auditory periphery [81], the harmonics are well represented in the auditory nerve firings (panel 1*b*), but when the two tones are presented together, the harmonics of the higher pitched tone tend to mask the harmonics of the lower pitched tone (a phenomenon referred to as two-tone masking) largely because the former are more intense than the latter due to the roll off in intensity with increasing frequency in natural sounds.

On the other hand, when the same tones are presented, but either the higher tone or the lower tone is occasionally presented 50 ms too early, as in panel 1*c*, from Hove *et al.* [82], the MMN is larger for the timing deviants in the lower pitched voice. As sounds propagate along the basilar membrane, the high frequencies enervate the basal end up to 10 ms sooner than the low frequencies enervate the apical end, but the low-voice superiority effect for time described here cannot be a consequence of this as this time difference is too short and the brain compensates for this difference, perceiving simultaneously presented high and low tones as simultaneous [83]. The origin of this effect in the inner ear depends rather on the harmonic structure of the tones, as can be seen by the results of passing these stimuli through the model of Ibrahim & Bruce [81]. In panel 1*d*, it can be seen that when the two tones come on simultaneously at 50 ms (top), the spike counts in the auditory nerve show a single abrupt onset across all frequency channels. When the lower pitched tone comes on too early at 0 ms (middle), there is spiking across the frequency range because its fundamental is low and its harmonics therefore cover the frequency range. In this case, there is no clear spike increase when the higher pitched sound enters at 50 ms and the sound is unambiguously represented as early. However, in the case that the higher tone is too early at 0 ms, there is spiking at this early time for frequencies at its fundamental and above, but a second clear spike increase is seen in the lower frequency range when the lower tone enters at 50 ms. Thus, the time representation of this stimulus is more ambiguous. These results show that the musical propensity to put the melody in the highest voice and the basic beat in the lowest voice originates in properties of the inner ear.

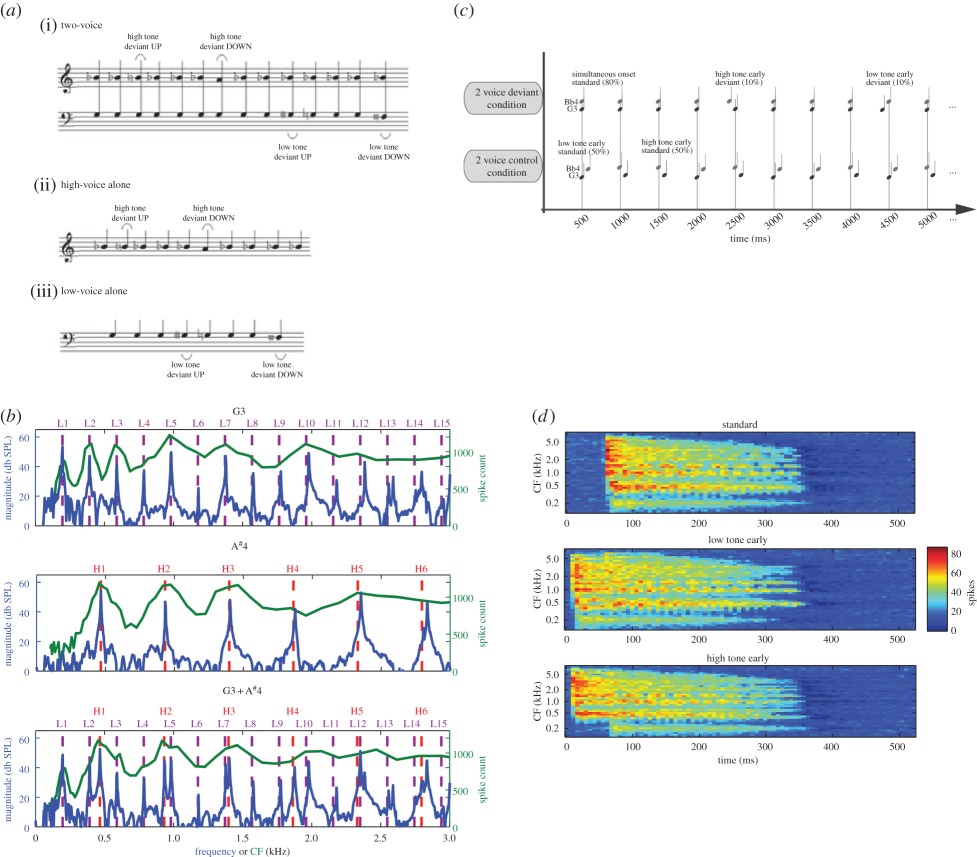

**Figure 2.**
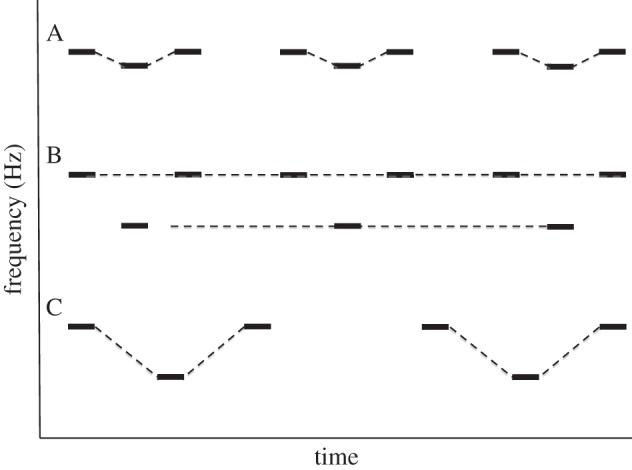
The effects of pitch proximity and tempo on determining the number of auditory objects in sequential streams of sounds. (A) When a higher tone repeats at a regular interval and a lower tone repeats at half the tempo of the higher tone, and they are arranged as in (A), all of the tones are perceived to come from a single sound source (as depicted by the dotted lines) and a gallop rhythm is heard. (B) When the higher and lower tones are sufficiently separated in frequency, they can no longer be integrated into a single stream. Two auditory objects are heard, one a repeating high tone and one a repeating low tone, and no gallop rhythm is perceived. This demonstrates that the auditory system expects a single sound source to remain reasonably consistent in pitch. (C) When the tempo of the sequence in (B) is slowed down, again the two pitches can be integrated into a single auditory object, and the gallop rhythm is heard again, consistent with the idea that the auditory system expects an auditory object to change pitch slowly. (Adapted from [41].)

Another aspect of ASA involves determining when to integrate successive sound events as emanating from one sound source (or stream) versus segregating them as emanating from different sound sources. A number of cues to streaming in ASA have been demonstrated (e.g. [36]), and Huron [42] has outlined how some of them relate to rules of musical composition. Huron's analysis applies to Western music, but it is likely that other musical systems are also greatly influenced by cues evolved for ASA. For example, one basic ASA cue for integration relates to *pitch proximity*; the frequency or pitch content of a source is expected to change little over small time periods, reflecting the fact that sound-emitting objects do not normally fluctuate rapidly in the frequency of the sounds produced. That this is a prominent cue in ASA was demonstrated with the gallop rhythm depicted in figure 2, A [41]. When the frequencies of the high and low tones are close, all of the tones are integrated into one auditory object, and a gallop rhythm can be heard. The larger the frequency distance between the high and low tones, the more likely it is that the pattern will be perceived as two auditory objects, one consisting of high tones and the other of low tones, in which case no gallop rhythm is heard (figure 2, B). Similarly, when the sequence is presented slowly, it is more likely that the tones with different frequencies will be integrated into one auditory object (figure 2, C), whereas at faster rates, the tones are more likely to separate into individual auditory objects.

Huron [42] showed that most of the Western rules of *voice leading* (how to compose polyphonic music) are a consequence of cues such as pitch proximity. For example, one set of rules states that when writing successive chords (e.g. in four-part harmony), where it is desirable for the listener to follow each part or stream (e.g. soprano, alto, tenor, bass), if it is possible, keep the same pitch in a particular part from chord to chord; if the pitch needs to change, move by the smallest pitch distance possible, and most importantly avoid large pitch changes. This enables people to perceive the successive tones from each part as coming from one auditory object and, therefore, to follow each part over time. Another rule states that it is not a good idea for the different parts to cross pitch so that, for example, the soprano part is higher than the alto part on one chord, but lower on the next chord. Again, the principle of pitch proximity dictates that under these conditions, listeners will be likely to confuse which pitches belong to which voice. In sum, the fit between compositional practice and the principles of ASA, and the fact that ASA is phylogenetically more ancient than human music, indicate that much of musical structure was not specifically selected for through evolutionary pressures for music, but rather that music conformed or adapted to a preexisting auditory system.

Some aspects of musical pitch, however, appear to be specific to music perception, such as the relation between sensory consonance/dissonance and feelings of pleasantness/unpleasantness, and the structure of musical tonality. According to Plomp & Levelt [84], two tones that are considered to sound pleasant together (consonant) have few harmonics between them that fall within critical bands, which is typically the result of their fundamental frequencies standing in small-integer ratios (e.g. octave 2 : 1; perfect fifth 3 : 2). On the other hand, tones that are perceived to sound unpleasant together (dissonant) stand in more complex ratios (e.g. major seventh 15 : 8; tritone 45 : 32) and have harmonics that fall within critical bands on the basilar membrane, creating the perception of beating and roughness. According to this theory, the perceptual differentiation of sensory consonance and dissonance derives directly from the structure of the basilar membrane. Assuming that there was no adaptive pressure for distinguishing consonant from dissonant tone combinations, this feature could be considered a spandrel of inner ear structure. Consistent with this notion is evidence that monkeys perceive the difference between sensory consonance and dissonance [85,86] even though they do not have music.

Interestingly, despite their ability to perceive the difference between consonance and dissonance, monkeys seem to have no preference for one over the other [33]. What seems to be special to human music, then, is a *preference* for consonance over dissonance, and the use of dissonance to create musical tension, and consonance to resolve that tension. Based on ideas articulated by Stumpf more than 100 years ago [87], McDermott *et al*. [88] proposed that the perception of consonance, defined as preference, was related to the extent to which all harmonics across the simultaneously presented sounds conformed to a harmonic template consisting of a fundamental frequency and harmonics at integer multiples of that fundamental. Experimentally, they showed that pleasantness has stronger relations to harmonicity than to roughness and beating. It is unknown whether monkeys base their discrimination of consonant and dissonant patterns on beating and roughness or on harmonicity, but it is possible that valenced harmonicity processing is unique to humans. It is clear that musical structure uses preexisting properties of the auditory system that give rise to the distinction between consonance and dissonance, but music appears to add emotional meaning to this distinction. The critical question, then, is whether this assignment of meaning is innate and was specifically selected for, making it a musical adaptation, or whether is it culturally derived. Studies of human infants are potentially informative in this regard, but the results are mixed. Several studies show preferences for consonance early in development [89–91], but it is unclear whether these early preferences are based on beating and roughness or on harmonicity, and whether they are learned or innate [92]. Furthermore, although it is often assumed that the perception of consonance and dissonance is similar around the world, there is limited evidence to support this assumption. Thus, it can be concluded that human music makes use of the species-general consonance/dissonance distinction, but that further research is needed to determine whether the differential assignment of emotional meaning is an adaptation for music or culturally derived.

More broadly than the consonance/dissonance distinction, musical pitch organization has a tonal structure, which dictates which pitch intervals (distances between tones) are used, the functions of different tones within musical scales, and how they are combined sequentially and simultaneously in composition and improvisation (e.g. see [93,94] for detailed descriptions of Western tonal pitch space). Just as there are many different languages in the world that share commonalities suggestive of innate biological constraints, there are many different musical systems in the world that share commonalities (e.g. [4,5,95–97]). Aspects of musical pitch structure that appear to be near universal across musical systems include octave equivalence (musical pitch has several perceptual dimensions, e.g. *chroma*, or notes of a scale, and *octave equivalence*, whereby pitches an octave apart are perceived to be similar and have common note names across octaves); the use of a small number of discrete pitches per octave (e.g. musical *scales*), which is likely a consequence of general memory limitations; and the use of more than one interval size (pitch distance) between notes of musical scales. The latter distinction enables each note of the scale to be related to the other notes in unique ways in terms of pitch relations [98,99]. Typically, one note (the tonic) is central, and each other note stands in a unique interval relation to the tonic and to the other notes. Collectively, these relations constitute the pitch space.

Critical questions concern how unique these properties are to human perception, and the extent to which they are the direct result of ASA and the basic structure of the auditory system, or whether they have cultural origins. Most of the properties of tonal pitch space noted above do not directly enhance the perception of auditory objects in the environment and are therefore unlikely to reflect direct adaptations for ASA. Furthermore, for the most part, they are not particularly useful for other auditory processing such as that needed for speech perception. And while the processing of tonal pitch space may rely on faculties such as memory and attention, these cannot fully explain the properties of tonal pitch space [97]. Tonal pitch space and the interval structure of scales appear to be relevant for music alone. Thus, one possibility is that tonal pitch space is a music-specific adaptation. Several genetic studies report that variation in musical ability has a strong genetic component ([100–105]; for a review, see [106]). However, this tells us little about whether there were selection pressures specifically for music. Although natural selection reduces genetic variability, highly polygenic adaptations, which would characterize music, are expected to show substantial genetic variability as a result of mutation-selection balance [107]. Additionally, the reported genetic differences might actually reflect variation in ASA ability as well, and may tell us nothing about music-specific adaptations. In terms of human development, infants and young children learn the specific pitch structure of the music in their environment without formal instruction, just as they learn the language in their environment, suggesting an innate ability to acquire this knowledge, although this ability may or may not be specific to music (e.g. [108–112]). A learning mechanism that was selected for one function but is used in a new domain is considered an exapted learning mechanism [1].

Conceiving of tonal pitch space as a music-specific adaptation faces the challenge of different musical systems having somewhat different tonal pitch spaces and the rapidity with which tonal pitch spaces change across time and when different musical systems come into contact, issues that apply equally to adaptationist arguments for language. Recent modelling of language acquisition and change suggests that it is not necessary, indeed very difficult, to postulate an innate universal grammar [29]. According to this view, rather than language being an evolutionary adaptation, it is a cultural creation moulded on preexisting perceptual and cognitive structures adapted for other purposes. It is possible that music behaves similarly and is a cultural creation based on preexisting features of the brain.

Interestingly, while different musical systems use somewhat different scales and have different tonal centres, certain intervals tend to be prominent across musical systems [113]. Recent work by Large and co-workers [114–116] shows that neural resonances in the auditory pathway induced by nonlinearities in the system give rise to the intervals prominent across musical systems and that models of such nonlinear oscillation easily learn properties of specific tonal pitch spaces. Thus, the emergence of musical intervals may, in fact, be a spandrel of basic properties of neural circuits. One difficulty with this argument is that such nonlinear neural circuits are also present in other species, raising the question of why these species have not developed tonal music. Without further research, a definite answer is impossible. However, it is possible that the potential for tonal pitch space perception is present in other species, but they lack other essential features such as sufficient memory capacity, a link between tonal pitch space and emotional meaning, a cultural means of sustaining such a complex system, or the motor skills to produce music. Indeed, octave equivalence, like the perceptual distinction between consonance and dissonance, has been found in monkeys, at least for simple tonal melodies [117], although non-human species in general have a greater propensity than humans to engage in absolute rather than relative pitch processing.

A further aspect of tonal pitch spaces is important with regard to their origins. Pitch space organization is related to meaning and emotion, as it enables the alternation of tension (moving away from the tonic) and relaxation (moving toward the tonic), and different scales in different musical systems are associated with different meanings. For example, music composed in the Western minor scale tends to convey sadness more than music composed in the major scale. Similarly, many Indian ragas are associated with different meanings and are meant to be played at different times and circumstances. Just as other species may perceive the distinction between consonance and dissonance but not show preferences in this regard, the mapping of meaning through tonal pitch space is a crucial aspect of human music, and the origin of this mapping must be part of any complete account of the origins of tonal pitch space.

## 4. Time processing and the origins of musical rhythm

Information about the timing of events plays a complementary role to spectral information in ASA [36]. For example, whether frequency component onsets are simultaneous or not is an important cue for determining whether they originate from the same source, as it is expected that onsets of components emanating from a single auditory object should begin at the same time. Conversely, components with non-simultaneous onsets will tend to be perceived as belonging to different auditory objects. This principle is central to musical structure. In cases where it is desirable for different simultaneous voices to fuse into a single percept with chordal quality, as in a barbershop quartet, various voices tend to have simultaneous onsets. On the other hand, in polyphonic music in which it is desirable for each part to be perceived as an independent voice, as in a fugue, each voice tends to change notes at different times [42]. As with a number of properties of spectral sound processing, such timing capabilities of the auditory system were likely adaptations for ASA, and musical structure has adapted to these preexisting adaptations rather than driving their existence.

Another basic principle of musical composition is to lay down the basic beat in the lowest pitched (bass) instruments. Recent research indicates that when two tones are presented simultaneously in a repeating sequence, listeners are better at detecting when the lower tone is occasionally presented 50 ms early (leaving the higher tone on time) compared with when the higher tone is presented 50 ms early (leaving the lower tone on time) [82]. Furthermore, modelling work suggests that this low-voice superiority effect for time originates in properties of the inner ear (see box 1) although the effect is probably sharpened higher in the auditory system [118,119]. As there is no obvious adaptive reason for this effect, it might simply be a non-adaptive consequence of the structure of the inner ear (spandrel). The important point with respect to music is that music is composed to conform to this preexisting feature of the auditory system.

As with tonal pitch space, aspects of musical rhythm appear to be specific to music (e.g. [8,120]). Language, for example, has temporal structure, but not the same requirement as music for regularity and temporal precision at the beat level. Musical rhythm has a number of aspects (e.g. [94,121]). The *rhythmic surface* consists of the sequence of event durations and silences that comprise the music. From this surface, the brain derives the *beat*, typically a regularly spaced sequence of pulses. That the beat is derived in the brain and not given directly in the stimulus is seen in beats that can be perceived even when there is no physical sound present but the surrounding context implies a beat at that time. EEG studies show brain signatures of such ‘felt’ beats (e.g. [122,123]). Beats can be mentally subdivided (usually into groups of 2 or 3) or every second or third beat can be perceived as accented, and these levels of beat structure form a metrical hierarchy. In humans, the beat is extracted effortlessly [124,125]. Furthermore, sensitivity to metre has been shown in young human infants [126–128].

One of the interesting aspects of musical behaviour is spontaneous movement to the beat of music [129]. Indeed, most people readily entrain their movements to the beat of music, using various effectors, across tempos from about 1 to 5 Hz. fMRI studies indicate that when listeners *perceive* musical metre, even in the absence of movement, a wide range of cortical and subcortical (premotor and supplementary motor cortex and basal ganglia) regions are activated [130–132]. Furthermore, when isochronous beat patterns are presented, EEG studies reveal that activation in the beta band (15–25 Hz) is modulated at the tempo of the beat [133,134]. Specifically, beta power decreases after each tone onset and rebounds in a predictive manner prior to the onset of the next beat, with the rebound delayed for slower tempos. Interestingly, this same pattern is observed in both auditory and motor regions when people simply listen to the beat, suggesting a strong connection between auditory and motor systems [122,133]. Furthermore, the influence appears to be bidirectional, in that when people move on either every second or third beat of an ambiguous rhythm pattern (one that can be interpreted as having different metrical structures such as a march or waltz), their movement influences the metrical interpretation of the auditory pattern [135].

Different timing mechanisms are present in the human brain. Neural circuits for duration-based (absolute) timing can be contrasted with beat-based timing, in which events occur at regular, predictable times [136,137]. Musical structure, of course, requires beat-based timing. Developmental and comparative studies are informative about the origins of the ability to perceive beat and metre, and the ability to entrain movements to a beat. With respect to non-human species, very few seem to entrain to a beat [32]. While there are no reports of motoric entrainment to an auditory beat in the wild, some vocal learning birds have demonstrated entrainment in captivity [31,32], and one mammal (sea lion) has been trained to move to the beat [138]. Despite these cases, this ability appears to be rare across non-human species and, even in cases where it is found, it requires considerable experience or training with humans and their music. Of course, many species produce rhythmic movements, and the advantage of locomotion was probably a major selective pressure for the development of rhythmic movement. But where humans appear to differ from most other species is in the connections between auditory and motor regions that support metrical perception and motor entrainment to an auditory beat [120]. Studies in non-human primates show that duration-based timing is universally present across primate species, but that only rudimentary beat-based timing is present in monkeys and chimpanzees [137]. Furthermore, the evidence suggests that in monkeys, sensorimotor connections for timing are stronger between vision and movement than between audition and movement [139,140], whereas the reverse is true for humans [141]. In line with this differentiation across primate species, although human infants are too motorically immature to precisely entrain to the beat [142], they do speed up their movements with increasing beat tempo [143]. Moreover, when bounced on either every second or third beat of an ambiguous rhythm pattern, bypassing their motoric immaturity, infants later prefer to listen to the pattern with accents corresponding to how they were bounced [127]. This indicates that motor influence on auditory perception is present in human infants and suggests that the privileged auditory–motor connections for beat and metre that, among primates, are unique to humans are present very early in human development.

Thus, it would appear that the ability for beat-based timing and the privileged connections between auditory and motor systems that enable entrainment to a beat evolved relatively recently within the primate lineage. The question, then, is whether beat-based timing was a music-specific adaptation or whether it emerged for other reasons. A comparison of tonal pitch space with beat-based timing and entrainment in this regard might be useful in addressing this question. Although tonal pitch space appears to be unique to humans, the particular pitch intervals used and their organization may originate in basic properties of nonlinear oscillators that characterize neural circuits. In this case, the neural basis of tonality would be widely conserved across species and an explanation is necessary for why humans exploited this feature to create music, whereas other species did not. On the other hand, beat-based timing ability and movement entrainment to an auditory beat appear to be substantially different in humans than in other primate species although a progression of ability in this regard can be seen in the primate lineage [137], and may rely on auditory motor circuits that are unique to humans [120]. Thus, it is possible that these capabilities are not easily explained by non-musical adaptations. The ability to entrain to an auditory beat of course enables individuals to synchronize their movements with others.

## 5. Social and emotional functions and the origins of music

In many cases, musical structure conforms to the properties of an auditory system that evolved for ASA, as discussed above. However, two central features of music cannot be explained completely by ASA, namely that music induces *emotional responses* in people and that music is an intensely *social activity*. The emotional and social aspects of music are probably closely related. With respect to *emotion*, music not only expresses emotion but it can induce emotions directly that can be measured physiologically (e.g. by changes in heart rate, galvanic skin responses, EEG and fMRI), behaviourally (e.g. tears) and by verbal reports of emotional experiences [144–147]. Common experience of music can, therefore, instill common emotional reactions in a group of people. This is probably why, even in modern society, people participate in music making or music listening in groups when the goal is to feel a common emotion and/or to work together to achieve a common goal. For instance, music is almost always present at important social functions such as weddings, funerals and parties. Fans chant to display their solidarity and offer encouragement at sporting events. Music is used in the military to encourage unity of purpose and to present a threatening front to the enemy.

Some properties of non-musical sounds can induce emotions across a range of species. For example, large menacing animals typically make low, loud sounds, and many species react to such sounds with fear [148]. Emotions can also be induced by unexpected events, and music exploits this basic mechanism as well [145,149,150]. Music exploits these emotional connections to sounds that are conserved across many species, but music appears to go beyond this basic emotional response to sound in using elaborate tonal systems (e.g. Western tonality and Indian ragas) that can express a myriad of emotions, many of which are hard to express verbally. Likewise, metrical structure provides a scaffold on which a variety of tempos and rhythmic patterns can induce a range of emotions from peacefulness to agitation and menace. Furthermore, the emotional impact of music in humans is seen early in infancy. For example, mothers sing lullabies to soothe infants and play songs to arouse them and interact playfully [16], and these have differential consequences for infants [151]. Emotional responses to music may be specific to humans and appear to be mediated by specialized physiological mechanisms. In humans, emotional responses to music are mediated by the dopamine system, such that music modulates activation in reward centres in the brain [146]. More physiological research is needed, but the apparent indifference of other primates to music [33] and very early responses in human infants suggest basic genetically driven differences in the physiology of neural pathways underlying the human emotional response to music and that of other primates. However, this question needs to be informed by more data across species.

With respect to social affiliation, after people move together in synchrony, they rate each other as more likeable, and they are more likely to cooperate than after moving asynchronously [152–156]. Because of its predictable beat, music provides an excellent scaffold for synchronized movement with others. Indeed, music and dance are intimately connected, and dance most often involves two or more people. It is notable that dancing is common during courtship, when strong social and emotional bonds are being formed. With respect to development, children who played a game together involving music were more likely to help each other than children who played a game together without music [157]. Furthermore, recent research indicates that infants as young as 14 months of age help an experimenter more (for example, by picking up items she ‘accidentally’ drops) if they were previously bounced to music in synchrony with her movements than if they were bounced at a different tempo [158]. Furthermore, this effect is specific to the person the infant bounced with and does not generalize to other people [159]. Thus, synchronous movement can have powerful effects on social affiliation and cooperation, can help define social groups and is effective very early in development. Indeed, an infant's experience of being rocked in their mother's arms while being sung to is potentially powerful in enhancing bonds between mother and infant. During adolescence, when the formation of social groups is very important, music is often used to help define individual and group identity [160].

Despite the universality and early emergence of entrainment effects (when motor immaturity of young children is bypassed) and associated affiliative consequences, motoric entrainment to an auditory beat has not been found in non-human species in the wild (although more research is needed), only a few species spontaneously engage in this behaviour when living with humans [32] and it is very difficult, if not impossible, to train this ability in those species that are genetically closest to humans [141]. Furthermore, there appear to be genetically driven physiological differences between human and non-human primates that underlie entrainment [120]. Thus, unlike many of the features of music that rest on adaptations for other functions such as ASA, emotional responses to music, entrainment and their affiliative consequences are candidates for music-specific adaptations.

Going back to Darwin [9], it has been proposed that musical behaviour evolved as an indicator of fitness, such that those with good rhythmic entrainment abilities, for example, would be more likely to attract mates [10]. This contention is consistent with the observation that, across a wide range of species, elaborate displays such as the peacock tail, which are potentially detrimental to survival by exposing the animal to predators and taking resources away from other activities that might increase survival, are often explained as signals of fitness to conspecifics [161]. According to this hypothesis, musical behaviour is an evolutionary adaptation such that the structure and production of music became more and more elaborate through competition as a display of the highest fitness. This view is not without challenges. A full discussion is beyond the scope of this paper, but the fact that both men and women produce music contrasts with the vast majority of such displays in other species, many of which are specific to males [162]. It is possible, however, that music is an outlier on this dimension, and both male and female humans engage in mate selection. Perhaps a more serious challenge is to explain why music is used across a range of situations that seemingly have little to do with mating, such as work songs, parental songs for infants and children's play songs.

Another proposal is that participating in joint music making increased group cohesion, cooperation and, therefore, the survival of individuals who were able to engage in music (e.g. [8,14,17,42]). Consistent with this view is evidence that, among primates, only in humans does music engage the dopamine reward system, and only in humans are there privileged connections between auditory and motor systems underlying beat and metrical processing. On the other hand, music is highly flexible, generative and changes rapidly over time, which pose particular challenges for an evolutionary theory of music. Furthermore, it is clear that, in large part, musical structure conforms to preexisting features of the auditory system, many of which evolved for ASA and are highly conserved across species, which strongly suggests that music is a cultural creation rather than an evolutionary adaptation. While these two views appear contradictory, they can be reconciled if a complex interaction between evolutionary and cultural processes is considered. For example, music may have originally emerged as a cultural creation made possible by preexisting adaptations related to ASA and other capabilities such as increased memory. However, if benefits arose through increased survival of those who engaged in music making, this could have exerted evolutionary pressure to enhance neural pathways by which music could activate emotional centres in the brain and to enhance pathways linking auditory and motor beat-based timing circuits. In turn, these neurally based adaptations could reinforce the cultural development and sustainability of musical behaviour, and perhaps explain why humans spend so much time and resources on music and why music is constantly changing.

## 6. Conclusion

In this paper, it is argued that both evolutionary adaptation and cultural creation probably played a role in the origins of music. Rather than focusing on an evaluation of different evolutionary versus cultural theories for musical origins, this paper considers various musical features and whether they were selected to enhance music specifically or whether they were adaptations for non-musical functions. This analysis shows that many aspects of musical pitch and timing structure conform to features of auditory processing needed for ASA. Given that ASA is much more ancient than music, is highly conserved across many species and is present early in development, it is concluded that, in large part, music has been designed to conform to features of ASA, rather than driving the nature of auditory processing. This lends support to the idea that music may have begun as a cultural creation, exapting preexisting features of the auditory system that had evolved for ASA. However, some aspects of music are not easily explained by ASA or other general capabilities such as increased memory and motor skills. These include emotional and social effects of music. It is possible that engaging in music conferred survival advantages, which in turn led to some music-specific adaptations. For example, the ability to perform beat-based timing and to entrain movements to a regular pulse appears to differ between humans and other primates, and to be supported by genetically driven brain connections that are present early in human development. Synchronous movement leads to increased group cohesion and to potential survival advantages for those who can participate. In this case, music may have conferred survival advantages that led to specific adaptations underlying behaviours such as entrainment, which had advantageous consequences such as social cohesion. Thus, music is likely to have a complex origin involving exaptation of traits evolved for other functions such as ASA, cultural creation and music-specific adaptations.
